# Estimation of individual animal SNP-BLUP reliability using full Monte Carlo sampling

**DOI:** 10.3168/jdsc.2020-0058

**Published:** 2021-03-19

**Authors:** H. Ben Zaabza, E.A. Mäntysaari, I. Strandén

**Affiliations:** Natural Resources Institute Finland (Luke), FI-31600 Jokioinen, Finland

## Abstract

Calculation of individual animal reliability of estimated genomic breeding value by SNP-BLUP requires inversion of the mixed model equations (MME). When the SNP-BLUP model includes a residual polygenic (RPG) effect, the size of the MME will be at least the number of genotyped animals (*n*) plus the number of SNP markers (*m*). Inversion of the MME in SNP-BLUP involves computations proportional to the cube of the MME size; that is, (*n* + *m*)^3^, which can present a considerable computational burden. We introduce a full Monte Carlo (MC) sampling-based method for approximating reliability in the SNP-BLUP model and compare its performance to the genomic BLUP (GBLUP) model. The performance of the full MC approach was evaluated using 2 data sets, including 19,757 and 222,619 genotyped animals selected from populations with 231,186 and 13.35 million pedigree animals, respectively. Genotypes were available in the data sets for 11,729 and 50,240 SNP markers. An advantage of the full MC approximation method was its low computational demand. A drawback was its tendency to overestimate reliability for animals with low reliability, especially when the weight of the RPG effect was high. The overestimation can be lessened by increasing the number of MC samples.

## Highlights

• Computing time for the full Monte Carlo (MC)-SNP-BLUP was less than for the exact genomic BLUP.
• The full MC-SNP-BLUP better approximated estimated breeding value reliability than an incomplete MCbased SNP-BLUP approach when the residual polygenic effect was high.
• The higher the exact reliability, the smaller the inflation.

## Graphical Abstract

**Figure:**
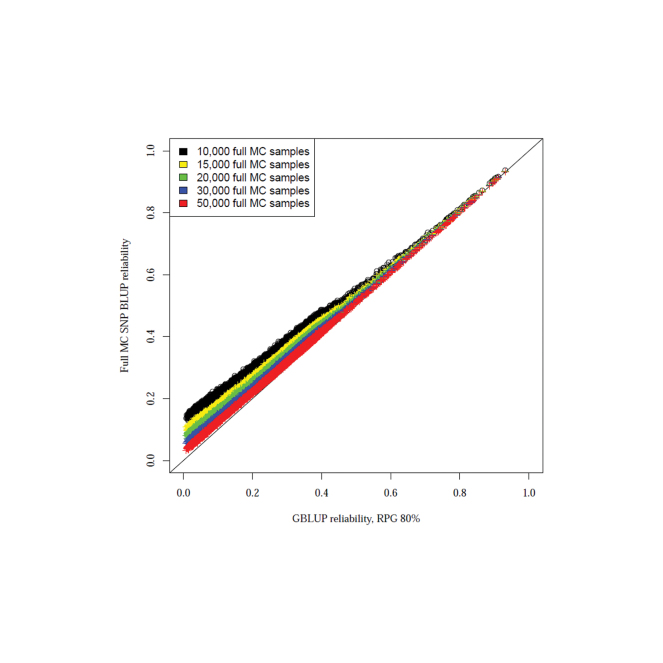
**Summary:** Reliabilities of estimated breeding values from a SNP-BLUP model can be calculated using elements of the inverse coefficient matrix of the mixed model equations. Computation of the inverse is not feasible for large data sets when the model has a residual polygenic (RPG) effect. We developed a full Monte Carlo (MC) samplingbased method for approximating reliabilities in the SNP-BLUP model with an RPG effect. Reliabilities obtained by the full MC approach were compared with the corresponding exact reliabilities obtained by the GBLUP model. The full MC approach provided good approximations to the exact values with only a small upward bias. The full MC approach is computationally efficient even for large data sets.

In the last decade, genomic selection has become the main source of genetic progress in dairy cattle breeding (Mäntysaari et al., 2020). Routine genomic evaluations in animal breeding are usually performed using genomic relationship-based BLUP (**GBLUP**) or random regression-based SNP-BLUP models. These 2 models are equivalent and, thus, yield equal EBV and prediction error variances (**PEV**) at the animal level, regardless of whether or not a residual polygenic (**RPG**) effect is fitted (Strandén and Garrick, 2009; Liu et al., 2016; Ben Zaabza et al., 2020a). Genetic variation cannot be completely explained by SNP markers because of the incomplete linkage disequilibrium between the SNP markers used and the QTL. The RPG effect models the proportion of genetic variance not captured by the SNP markers.

The PEV can be used to obtain the accuracy of EBV or its square—the reliability (Schaeffer, 2019). The calculation of EBV reliability requires elements from the inverse of the coefficient matrix of the mixed model equations (**MME**). In GBLUP models, the inverse of the genomic relationship matrix (**G**) has to be computed as well. The **G** matrix is a dense matrix of the size of the number of genotyped animals (*n*) without a known simple structure (Meyer et al., 2013). Thus, the **MME** and **G** inverse matrices need to be explicitly computed with a cubic computational cost O(*n*^3^) of the number of genotyped animals. This is not tenable for very large values of *n*. The SNP-BLUP model requires no **G**^−1^ matrix and the MME size is bounded by the number of markers (*m*). When *n* > *m*, the SNP-BLUP model yields fewer equations to solve than the GBLUP model. However, although low-cost SNP arrays with approximately 50,000 genome-wide SNPs are often used today for most livestock species, even a 700,000-SNP chip array can be used (Meuwissen et al., 2013). With such a high-density SNP chips available, calculating the inversion of the MME in SNP-BLUP can therefore present, at some point, a considerable computational burden.

When the RPG effect is included in the SNP-BLUP model, the computational cost of inverting the MME in SNP-BLUP increases from O(*m*^3^) to O[(*n* + *m*)^3^]. Reducing the size of the MME can help decrease some of the computational costs. Ben Zaabza et al. (2020a,b) presented a Monte Carlo (**MC**) approach for the RPG effect in an SNP-BLUP model, in which the number of RPG effects was reduced from *n* to the number of MC samples. In this article, we describe a full MC-based SNP-BLUP approach that extends the approach in Ben Zaabza et al. (2020a) to reduce the computational requirements in the calculation of SNP-BLUP genomic reliabilities. We compare the performance of the fully MC-based SNP-BLUP approach to GBLUP where the model has both SNP marker effects and RPG effects.

The SNP-BLUP model with an RPG effect can be expressed as (Ben Zaabza et al., 2020a)

[1] $$\text{y}=1_{n}\mu +{\text{Zg}}_{\text{R}}+\text{e},$$

where **y** is an *n* × 1 vector of observations; *n* is the number of phenotypic records; **1***_n_* is a vector of *n* ones; *μ* is the unknown overall mean; $\text{Z}=\left[\begin{array}{cc} \sqrt{\left(1-w\right)}{\text{Z}}_{c} & \sqrt{w\text{L}} \end{array}\right],$ where *w* is the proportion of RPG, **Z***_c_* is a centered and scaled genotype marker matrix of order *n* × *m*, the square matrix **L** of size *n* is the Cholesky decomposition of the pedigree-based relationship matrix of genotyped animals **A**_22_; that is, **A**_22_ = **LL′**; and **g*_R_*** is an *m_n_* × 1 vector of unknown genetic effects, *m_n_* = *m* + *n*. The coefficients in the **Z***_c_* matrix are genotypes at the SNP markers: $\frac{1}{S_{k}}\left(0-2p_{k}\right)$, $\frac{1}{S_{k}}\left(1-2p_{k}\right)$, and $\frac{1}{S_{k}}\left(2-2p_{k}\right)$ for genotypes B_1_B_1_, B_1_B_2_, and B_2_B_2_, respectively, where *p_j_* is the allele frequency of B_2_ at locus, j = 1, …, *m*. Commonly used scaling factors are $S_{k}=\sqrt{\sum_{l=1}^{m}2p_{l}\left(1-p_{l}\right)}$ and $S_{k}=m\sqrt{2p_{k}\left(1-p_{k}\right)}$ often called VanRaden (2008) methods 1 and 2, respectively. Base population allele frequencies are preferably used for *p_j_*. It is assumed that ${\text{g}}_{R}\sim N\left(0,{\text{I}}_{m_{n}}\sigma_{u}^{2}\right)$ and $\text{e}\sim N\left(0,\text{R}\sigma_{e}^{2}\right)$ where $\sigma_{u}^{2}$ is the genetic variance, ${\text{I}}_{m_{n}}$ is an identity matrix, and $\sigma_{e}^{2}$ is the residual variance. The diagonal matrix **R** has elements ${\text{R}}_{ii}=\frac{1}{w_{i}}$, where *w_i_* is the weight for observation *i*. Note that this model assumes $Var\left({\text{Zg}}_{R}\right)=\text{Z}{\text{Z}}^{\prime }\sigma_{u}^{2}={\text{G}}_{w}\sigma_{u}^{2},$ where $G_{w}=\left(1-w\right){\text{Z}}_{c}{{\text{Z}}^{\prime }}_{c}+w{\text{A}}_{22}$ is the genomic relationship matrix with an RPG proportion of *w*.

Ben Zaabza et al. (2020a) presented a model in which MC samples were used instead of the Cholesky decomposition **L** for the RPG effect in [1]. Their MC-SNP-BLUP model can be extended to use MC samples for the SNP marker effects as well. We call this SNP-BLUP, which uses MC sampling for all genetic effects as full-MC-SNP-BLUP. The full-MC-SNP-BLUP model can be written as

[2] $$\text{y}=1_{n}\mu +\text{Us}+\text{e},$$

where **s** is an *n_MC_* × 1 vector of random pseudo-genetic effects, *n_MC_* is the number of MC samples, and **U** is an *n* × *n_MC_* matrix of the MC samples. The random effects are assumed to be normally distributed as $\text{s}\sim N\left(0,\text{I}\sigma_{u}^{2}\right),$ and $\text{e}\sim N\left(0,\text{R}\sigma_{e}^{2}\right).$ Each column *i* in the regression matrix $\text{U}=\left[{\text{u}}_{1}\cdots {\text{u}}_{n_{MC}}\right]$ is an MC simulated sample ${\text{u}}_{i}={\text{Zg}}_{i}+{\text{a}}_{i},$ where the vectors **g***_i_* and **a***_i_* can be sampled from their assumed distributions: g~N[0,I(1-w)] and $\text{a}\sim N\left(0,w{\text{A}}_{22}\right).$ Hence, **u***_i_* is a sample from $N\left(0,{\text{G}}_{w}\right).$ Note that the genetic variance $\sigma_{u}^{2}$ is a variance parameter for the genetic effects of the model such that the sampling can be performed without it. For more details on the MC sampling of the RPG effect vector **a**, see Ben Zaabza et al. (2020a).

The MME corresponding to the full-MC-SNP-BLUP model [2] can be written as

[3] $$\left[\begin{array}{cc} {1^{\prime }}_{n}{\text{R}}^{-1}1_{n} & {1^{\prime }}_{n}{\text{R}}^{-1}\text{U}\\ \text{U}’{\text{R}}^{-1}1_{n} & \text{U}’{\text{R}}^{-1}\text{U}+\text{I}\lambda \end{array}\right]\left[\begin{array}{c} \hat{\mu }\\ \hat{\text{s}} \end{array}\right]=\left[\begin{array}{c} {1^{\prime }}_{n}{\text{R}}^{-1}\text{y}\\ \text{U}’{\text{R}}^{-1}\text{y} \end{array}\right],$$

where $\lambda =\frac{\sigma_{e}^{2}}{\sigma_{u}^{2}}.$ Denote the coefficient matrix of this MME by **C***_u_* and its inverse matrix elements as ${\text{C}}_{u}^{-1}=\left[\begin{array}{cc} {\text{C}}_{\text{u}}^{\mu \mu } & {\text{C}}_{\text{u}}^{\mu s}\\ {\text{C}}_{\text{u}}^{s\mu } & {\text{C}}_{\text{u}}^{ss} \end{array}\right].$ Approximate reliability (r^2^) for EBV of animal *j* can be calculated as:

$$r_{j}^{2}=1-\lambda \frac{{\text{PEV}}_{j}}{\sigma_{j}^{2}},$$

where **PEV***j* is the prediction error variance and $\sigma_{j}^{2}$ the genetic variance for the *j*th individual. Here, **PEV***j* can be calculated as ${\text{PEV}}_{j}={\text{t}}_{j}{\text{C}}_{u}^{ss}{t^{\prime }}_{j},$ where **t***_j_* is equal to the *j*th row in **U**. The animal genetic variance $\sigma_{j}^{2}$ is the diagonal element *j* in the MC approximate genomic relationship matrix **UU′**. Note that instead of using the diagonal elements of **UU′** for $\sigma_{j}^{2},$ it is possible to compute and use the exact value. The exact value is $\left(1-w\right){\left\{{\text{Z}}_{c}{{\text{Z}}^{\prime }}_{c}\right\}}_{jj}+w\left(1-F_{j}\right),$ where *F_j_* is the inbreeding coefficient of animal *j*.

We applied the method to 2 data sets to demonstrate the efficiency of the proposed approximation. Our first data set (Data1) comprised genomic and pedigree information in Finnish Red dairy cattle. The pedigree included 231,168 animals. Genotypes were available for 19,757 animals. Because SNP-BLUP is computationally less demanding than GBLUP when the number of genotyped animals is greater than the number of SNP markers, the number of markers in Data1 was reduced to 11,729 SNPs by taking every fourth marker. The second data set (Data2) comprised genomic and pedigree information from a multibreed Irish beef cattle carcass conformation evaluation. Genotypes for 50,240 SNP markers were available for 222,619 animals. The pedigree included up to 13.35 million animals. A summary of Data2 is given in Mäntysaari et al. (2017).

Performance examination of the full-MC-SNP-BLUP reliability approximation involved 2 steps. First, a standard GBLUP model was used to calculate exact GBLUP model reliabilities. Second, the full-MC-SNP-BLUP model was used to calculate approximated reliabilities under 10 different MC sample sizes (10,000, 15,000, 20,000, 30,000, 40,000, 50,000, 60,000, 70,000, and 90,000). Reliabilities for the 2 approaches were computed using 3 RPG proportions (20, 50, and 80%). Performance statistics included correlation, maximum difference, and mean-squared error (**MSE**) between the exact GBLUP reliability and the full-MC-SNP-BLUP approximated reliability. We fitted the linear regression from the true GBLUP reliability on that from the full-MC-SNP-BLUP to investigate inflation (or deflation) in the approximated reliability. Computational performance was based on wall clock time at selected computing steps.

Table 1 gives statistics on GBLUP versus full-MC-SNP-BLUP for Data1 and Data2. For Data1, correlations between the reliabilities from the true and approximation methods ranged from 0.997 to 1.000. The correlation was lowest with a small MC sample size and high RPG weight. For example, the lowest correlation (0.997) was observed with an MC sample size of 10,000 and an RPG proportion of 80%. This scenario had the largest MSE (675 × 10^−5^) and the largest maximum difference (0.14).

**Table 1:** Correlation (r), maximum difference (max), and mean-squared error (MSE) between reliabilities from genomic BLUP and full Monte Carlo-SNP-BLUP models under different numbers of Monte Carlo samples (N_MC_) and residual polygenic proportions (*w*) in analysis of data sets 1 and 2^1^

N_MC_	*w*	Data 1					Data 2				
		r	max	MSE (×10^−5^)	b_0_	b_1_	r	max	MSE (×10^−5^)	b_0_	b_1_
10,000	0.20	0.997	0.10	231	−0.11	1.12	0.993	0.37	6,623	−0.85	1.93
	0.50	0.997	0.13	455	−0.13	1.15	0.989	0.46	11,645	−1.05	2.15
	0.80	0.997	0.14	675	−0.15	1.17	0.986	0.52	16,006	−1.19	2.31
20,000	0.20	0.999	0.06	66	−0.07	1.06	0.998	0.22	2,308	−0.37	1.41
	0.50	0.998	0.07	124	−0.09	1.08	0.996	0.29	4,401	−0.46	1.51
	0.80	0.999	0.08	188	−0.10	1.08	0.995	0.33	6,341	−0.51	1.58
30,000	0.20	0.999	0.04	29	−0.03	1.04	—	—	—	—	—
	0.50	0.999	0.06	58	−0.04	1.05	—	—	—	—	—
	0.80	0.999	0.06	86	−0.05	1.06	—	—	—	—	—
40,000	0.20	—	—	—	—	—	0.999	0.12	690	−0.17	1.19
	0.50	—	—	—	—	—	0.999	0.17	1,379	−0.21	1.24
	0.80	—	—	—	—	—	0.999	0.19	2,057	−0.24	1.27
50,000	0.20	0.999	0.03	11	−0.02	1.02	—	—	—	—	—
	0.50	0.999	0.04	21	−0.02	1.03	—	—	—	—	—
	0.80	1.000	0.04	33	−0.003	1.03	—	—	—	—	—
60,000	0.20	—	—	—	—	—	1.000	0.09	327	−0.11	1.25
	0.50	—	—	—	—	—	0.999	0.12	664	−0.14	1.16
	0.80	—	—	—	—	—	0.999	0.14	1,001	−0.15	1.18
70,000	0.20	—	—	—	—	—	1.000	0.07	243	−0.10	1.11
	0.50	—	—	—	—	—	0.999	0.11	499	−0.12	1.13
	0.80	—	—	—	—	—	0.999	0.12	757	−0.15	1.18

1 Where b_0_ = intercept and b_1_ = slope.

The largest relative decrease in MSE was observed when the number of MC samples was increased from 10,000 to 20,000 (Table 1). Increasing the MC sample size beyond 20,000 led to only a slight decrease in MSE and the maximum difference between full-MC-SNP-BLUP and GBLUP model reliabilities. The MSE values, which reflect accuracy or closeness, were consistent with the correlations, which reflect the association. In fact, the higher the observed correlation, the smaller the MSE. The results reaffirmed the association of the MC sampling sizes with the MSE and maximum difference estimates previously reported in Ben Zaabza et al. (2020a). In their study, MC sampling was used only for the RPG effect, and so it was logical that the higher the RPG weight, the greater the inflation in reliability.

We compared the MSE estimates obtained in this study to those reported in Ben Zaabza et al. (2020a). Note that in our full-MC-SNP-BLUP approach, the size of the MME is determined by the number of MC samples, but in the MC-SNP-BLUP by Ben Zaabza et al. (2020a), the MME size was determined by the number of markers plus the number of MC samples. For example, in full-MC-SNP-BLUP with 20,000 MC samples, we generated and made an MME of size 20,000, when the full model size would have been 11,729 SNP markers + 19,757 genotyped animals. In the MC-SNP-BLUP approach, 20,000 MC samples were used to generate only the RPG effect (19,757 genotyped animals), which made an MME of size 31,729. Given that the computing time to invert the MME coefficient matrix increases in cubic terms of its size, we compared the performance of these approaches at equal MME sizes.

The MSE (66 × 10^−5^ vs. 81 × 10^−5^) reached by full-MC-SNP-BLUP (20,000 MC samples) was similar to that obtained by MC-SNP-BLUP (10,000 MC samples) with an RPG weight of 20% for Data1 (Table 1). When the RPG proportion was increased to 50%, the MSE achieved by the full-MC-SNP-BLUP approach (20,000 MC samples) was much lower, 124 × 10^−5^, than that achieved by MC-SNP-BLUP, 1,822 × 10^−5^ (10,000 MC samples). The relative difference was even larger when the RPG weight was 80% (188 × 10^−5^ vs. 8,280 × 10^−5^). These results suggest that the full-MC-SNP-BLUP model approximation is less sensitive, in terms of MSE, to changes from low to high RPG weight than the MC-SNP-BLUP approach at the same MME sizes.

Table 1 shows the intercept (b_0_) and slope (b_1_) of the regression of correct reliability from GBLUP on approximation by full-MC-SNP-BLUP for Data1. An unbiased approximation method would be expected to give a null intercept and a slope equal to 1. Although the regression of GBLUP on full-MC-SNP-BLUP gave estimates of b_0_ close to 0 and b_1_ close to 1, all scenarios led to inflated reliability because the slopes were slightly greater than 1. The inflation decreased as the MC sample size increased or the RPG weight decreased, which was also observed by MC-SNP-BLUP using the same data (Ben Zaabza et al., 2020a). However, the full-MC-SNP-BLUP approach appeared to produce intercept and slope estimates smaller than those obtained by MC-SNP-BLUP, especially with an RPG weight larger than 20%. For example, in the scenario with an 80% RPG weight, the slope was 1.70 with MC-SNP-BLUP (10,000 MC samples) but reduced to 1.08 with full-MC-SNP-BLUP (20,000 MC samples). This was partly a consequence of comparing equal-size MME results where the number of MC samples used for approximating the RPG effect was larger in full-MC-SNP-BLUP than in MC-SNP-BLUP. On the other hand, the SNP marker effects were accounted for without MC sampling in MC-SNP-BLUP but they were approximated with MC samples in full-MC-SNP-BLUP. However, the results suggest that when the same number of MC samples is used to approximate covariance structure between animals, the simulated genomic-based covariance structure is closer to the true one than the simulated pedigree-based covariance structure because the number of SNP markers is less than the number of RPG effects.

Correlations between the exact GBLUP and approximate full-MC-SNP-BLUP model reliabilities for Data2 are in Table 1. Increasing the number of MC samples increased the correlation between the model reliabilities, which became clearer as the RPG proportion increased. When the number of MC samples was 10,000 or more, the full-MC-SNP-BLUP approach gave high correlations (>0.986) in all scenarios, but the size of bias for large RPG weights was still affected by the number of MC samples. For example, with full-MC-SNP-BLUP and an RPG weight of 80%, increasing the MC sample size from 10,000 to 60,000 reduced the MSE by up to 16 times. In general, increasing the number of MC samples further to 70,000 decreased the MSE only slightly under all RPG weights. It is worthwhile noting that to achieve the same level of unbiasedness in both data sets, the number of MC samples required for Data2 was about 6 times that for Data1. For example, the MC sample size needed to achieve an MSE of 231 × 10^−5^ and a maximum difference of 0.1 was 10,000 when analyzing Data1. However, more than 60,000 MC samples were needed to attain the same MSE with Data2. Note that Data1 was a single breed population, whereas Data2 included several breeds. This indicates that the goodness of the approximation may be influenced by several factors, such as the number of genotyped animals, structure of the population, heritability of the trait, and the trait's architecture (e.g., Ben Zaabza et al., 2020a).

We compared the analysis of Data2 between the full-MC-SNP-BLUP and MC-SNP-BLUP results at about equal MME sizes, as was done for Data1. Because Data2 had 50,240 markers, about 50,000 more MC samples were allowed in the full-MC-SNP-BLUP than in the MC-SNP-BLUP model when comparing the models. The MSE values with full-MC-SNP-BLUP were smaller than those obtained by MC-SNP-BLUP in all scenarios when the RPG proportion was greater than 20%, which was observed for Data1 as well. For example, with Data2, the MSE associated with full-MC-SNP-BLUP (70,000 MC samples) was smaller than observed with MC-SNP-BLUP (20,000 MC samples) using an RPG weight of 80% (757 × 10^−5^ vs. 3,142 × 10^−5^).

The regression of the reliabilities obtained from GBLUP on those approximated by full-MC-SNP-BLUP indicated a higher degree of correspondence than that between GBLUP and MC-SNP-BLUP. This became clearer, the larger the RPG weight. For example, for an MC sample size of 60,000, the slope was 1.16 (intercept −0.14) and 1.18 (intercept −0.15) for RPG weights of 50% and 80%, respectively, when analyzing Data2 using the full-MC-SNP-BLUP method. However, for an MC sample size of 10,000, the slopes (intercepts) corresponding to these RPG weights were 1.26 (−0.24) and 1.70 (−0.63), respectively, for Data2 using the MC-SNP-BLUP method. Nevertheless, the full-MC-SNP-BLUP approach tended to overestimate the reliabilities (data not shown). Furthermore, the lower the exact GBLUP reliability, the larger the overestimation.

The full-MC-SNP-BLUP computations used MC sampling to approximate the denominator $\sigma_{j}^{2}.$ However, it is possible to compute the exact value as stated in the section Full MC-SNP-BLUP Derivations. We tested the exact $\sigma_{j}^{2}.$ values in the full-MC-SNP-BLUP model calculations. However, the use of the exact $\sigma_{j}^{2}.$ decreased the accuracy (higher maximum difference and MSE), especially for RPG higher than 20%. Similar results were obtained with the MC-SNP-BLUP approach (Ben Zaabza et al. 2020a).

Table 2 gives the computing times for different steps in the calculation of reliability using full-MC-SNP-BLUP for Data1 and Data2, respectively. The computing times are averages over the used RPG weights. The full-MC-SNP-BLUP method involved 4 steps: making the MC samples for the marker effect, making the MC samples for the RPG effect, making the MME coefficient matrix, and inverting the MME matrix. Table 2 clearly shows that the computing times increased along with an increase in the number of MC samples, which would be expected, because the number of MC samples increases the number of computations in all the steps.

**Table 2:** Computing time (wall clock, in minutes) for calculating model reliability in full Monte Carlo (MC)-SNP-BLUP model under different MC sample sizes^1^

Computing step^2^	Sample size						
	10,000	15,000	20,000	30,000	50,000		
Data set 1							
MC-marker-effects	14	21	30	43	77	—	—
MC-RPG	32	46	66	99	163	—	—
Making MME	11	22	40	92	261	—	—
Inversion	7	26	54	170	565	—	—
Total	99	164	254	520	1,575	—	—
							
	5,000	10,000	20,000	40,000	60,000	70,000	90,000
Data set 2							
MC-marker-effects	5	10	18	36	54	61	82
MC-RPG	9	18	34	77	113	114	155
Making the MME	0.42	1.5	6	22	48	66	109
Inversion	0.02	0.13	1	7	21	34	73
Total	36	52	88	193	323	388	586

1 Data sets 1 and 2 had 19,757 and 222,619 genotyped animals, respectively, and 11,729 and 50,240 SNP.

2 MC-marker-effects = MC sampling of marker effects; MC-RPG = MC sampling of residual polygenic effects; Making MME = making the mixed model equations (MME); Inversion = inversion of the MME; and Total = total computing time.

For Data1, the time required for MC sampling of the marker effects was often lowest among the computing steps (Table 2). The computing times required for MC sampling of the RPG effect, on the other hand, were more expensive than those of the marker effects. However, both of these steps showed a linear increase in computing time with the number of MC samples. In contrast, the computing time for making the MME is roughly proportional to $n{\left(n_{MC}\right)}^{2}.$ For instance, increasing the number of MC sample from 10,000 to 50,000 increased the computing time by a factor of 24, which is close to the squared ratio of the MME sizes; that is, (50,000/10,000)^2^. Computing time to invert the MME is roughly proportional to ${\left(n_{MC}\right)}^{3}.$ Thus, for Data1, it was more expensive computationally to invert the MME than to make the MME when *n_MC_* exceeded the number of genotyped animals (19,757). For Data2, the number of genotyped animals *n* was always higher than the number of MC samples *n_MC_* (Table 2). Consequently, making the MME took more time than inverting the MME in all scenarios, accounting for 3% (1.5 min) to 19% (109 min) of the total computing time. Thus, it can be expected that, for large data sets, making the MME can be computationally more demanding than inverting the MME coefficient matrix.

Note that the relative differences between the total computing time and the 4 steps shown in Table 2 are due to the calculation of ${\text{t}}_{j}{\text{C}}_{u}^{ss}{{\text{t}}^{\prime }}_{j}.$ Computing this product in 2 steps from left to right for every individual requires $O\left[n{\left(n_{MC}\right)}^{2}\right]$ multiplications. In the Data1 analyses, *n_MC_* was often larger than *n*, but in Data2 analyses, *n_MC_* was always less than *n*; thus, the increase in *n_MC_* had a larger effect on computing time using Data1 than Data2. The time required for the calculation of the product ${\text{t}}_{j}{\text{C}}_{u}^{ss}{{\text{t}}^{\prime }}_{j}$ decreased from 35% of the total for 10,000 MC samples to 32% of the total for 50,000 MC samples with Data1. With Data2, the proportional computing time for this product decreased from 43% for 10,000 MC samples to 28% for 90,000 MC samples.

The computing times for calculating model reliability in genomic relationship matrix-based GBLUP are given in Table 3. In the analysis of Data1, the total computing time required by GBLUP was less than by full-MC-SNP-BLUP when the number of MC samples was more than 30,000. For Data2, the full-MC-SNP-BLUP approach always needed less time than GBLUP, and the reduction in total computing time was 81% (2,504 min) even with the largest MC sample size (90,000 MC samples). In GBLUP, most of the computing time was spent in inverting the 2 matrices (**G** and MME coefficient matrix). These 2 matrix inversions accounted for 28% (71%) of the total computing time for Data1 (Data2). With full-MC-SNP-BLUP, the computing time for matrix inversion depended on the number of MC samples and took at most 36% (12%) for Data1 (Data2). Thus, it is likely that with larger numbers of genotyped animals, the GBLUP approach will become computationally unfeasible due to the time needed for matrix inversion; in full-MC-SNP-BLUP, this time can be kept acceptable by controlling the number of MC samples.

**Table 3:** Computing times (wall clock, in seconds or minutes) for calculating individual animal reliability in genomic BLUP^1^

Computing step^2^	Data set 1 (s)	Data set 2 (min)
Making **G**	53	276
Inverting **G**	50	1,116
Making MME	2	216
Inverting MME	52	1,092
Total	365	3,090

1 Reported times are averages over 3 analyses using different residual polygenic weights.

2 Making **G** = during making the genomic relationship matrix; Inverting **G** = inversion of the **G** matrix; Making MME = making of the mixed model equations (MME); Inverting MME = inversion of the MME; and Total = total computing time.

The reliabilities of the SNP-BLUP models can be approximated satisfactorily by using a full MC-based sampling method. The correlation between the correct reliabilities calculated by GBLUP and those approximated by the full-MC-SNP-BLUP model approached unity in all studied scenarios. The computing time required to invert the MME coefficient matrix was roughly in the proportion ${\left(n/n_{MC}\right)}^{3}$ for GBLUP over the full-MC-SNP-BLUP model. The approximation method presented in this study is computationally efficient and thus is applicable for large-scale problems involving many genotyped animals and SNP markers.
